# Simultaneous evaluation of antibodies that inhibit SARS-CoV-2 variants via multiplex assay

**DOI:** 10.1172/jci.insight.150012

**Published:** 2021-08-23

**Authors:** Ester Lopez, Ebene R. Haycroft, Amy Adair, Francesca L. Mordant, Matthew T. O’Neill, Phillip Pymm, Samuel J. Redmond, Wen Shi Lee, Nicholas A. Gherardin, Adam K. Wheatley, Jennifer A. Juno, Kevin J. Selva, Samantha K. Davis, Samantha L. Grimley, Leigh Harty, Damian F.J. Purcell, Kanta Subbarao, Dale I. Godfrey, Stephen J. Kent, Wai-Hong Tham, Amy W. Chung

**Affiliations:** 1 Department of Microbiology and Immunology, The Peter Doherty Institute for Infection and Immunity, The University of Melbourne, Melbourne, Victoria, Australia.; 2 The Walter and Eliza Hall Institute of Medical Research, Melbourne, Victoria, Australia.; 3 Australian Research Council Centre for Excellence in Convergent Bio-Nano Science & Technology, The University of Melbourne, Parkville, Victoria, Australia.; 4 Australian Research Council Centre of Excellence for Advanced Molecular Imaging, The University of Melbourne, Melbourne, Victoria, Australia.; 5 WHO Collaborating Centre for Reference and Research on Influenza, The Peter Doherty Institute for Infection and Immunity, Melbourne, Victoria, Australia.; 6 Department of Medical Biology, The University of Melbourne, Melbourne, Victoria, Australia.

**Keywords:** COVID-19, Immunoglobulins

## Abstract

The SARS-CoV-2 receptor binding domain (RBD) is both the principal target of neutralizing antibodies and one of the most rapidly evolving domains, which can result in the emergence of immune escape mutations, limiting the effectiveness of vaccines and antibody therapeutics. To facilitate surveillance, we developed a rapid, high-throughput, multiplex assay able to assess the inhibitory response of antibodies to 24 RBD natural variants simultaneously. We demonstrate how this assay can be implemented as a rapid surrogate assay for functional cell-based serological methods to measure the SARS-CoV-2 neutralizing capacity of antibodies at the angiotensin-converting enzyme 2–RBD (ACE2-RBD) interface. We describe the enhanced affinity of RBD variants N439K, S477N, Q493L, S494P, and N501Y to the ACE2 receptor and demonstrate the ability of this assay to bridge a major gap for SARS-CoV-2 research, informing selection of complementary monoclonal antibody candidates and the rapid identification of immune escape to emerging RBD variants following vaccination or natural infection.

## Graphical abstract

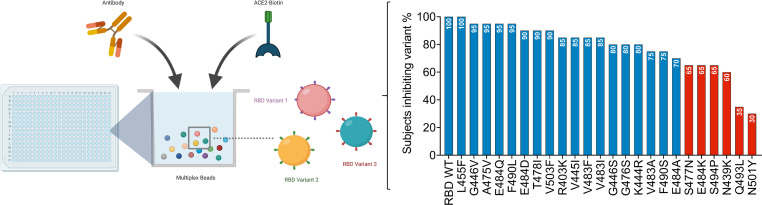

## Introduction

SARS-CoV-2 was first identified in late 2019 in Wuhan, China (1, 2), and has since become an ongoing global public health emergency. COVID-19 is the clinical syndrome associated with SARS-CoV-2 infection, which is characterized by a respiratory syndrome with a variable degree of severity. To date millions of deaths have been reported (3), with the current pandemic not only threatening public health but also adversely affecting economies worldwide. As we enter the second year of the SARS-CoV-2 pandemic, and promising vaccines are rolled out (4, 5), the ongoing transmission, along with the emergence of new variants with multiple mutations at key residues in the spike glycoprotein (6–8), have resulted in the continued implementation of stringent public health measures to control infection in many parts of the world.

SARS-CoV-2 enters host cells via the angiotensin-converting enzyme 2 (ACE2) receptor (9–11). Binding to ACE2 and entry to the host cell are facilitated via the spike, a homotrimeric transmembrane envelope glycoprotein, which consists of the binding (S1), and transmembrane fusion (S2), domains that are processed from the polyprotein precursor at a polybasic furin cleavage site (9, 12). Key to this virus–host interaction is the receptor-binding domain (RBD), which lies within the S1 subunit and is critical for binding to ACE2 receptors on target cells (11). Numerous studies have demonstrated that potent neutralizing antibodies (NAbs) that recognize the RBD are consistently elicited following infection (13–16) and account for most plasma neutralizing activity and inhibition of RBD-ACE2 binding (13, 17). Antibodies targeting the RBD account for approximately 90% of the neutralizing activity present in SARS-CoV-2 immune sera (17) and therefore have great potential to be clinically useful in the treatment and prevention of SARS-CoV-2 infection. The RBD of SARS-CoV-2 therefore represents a key target for the development of vaccine-elicited humoral immunity, and eliciting potent NAbs capable of blocking ACE2 binding remains a key feature of the development of effective vaccines and antibody therapies.

As for any viral outbreak, of great concern is the emergence of gain-of-function variants, which facilitate viral infectivity, transmissibility, or neutralizing antibody escape. Since the beginning of the pandemic, SARS-CoV-2 genomic sequencing has identified a number of variants, revealing a modest rate of evolution of the viral genome (18, 19). This information has been made available through public repositories, such as the Global Initiative on Sharing All Influenza Data (GISAID; ref. 20), which have been critical to monitoring the epidemiology of the virus and informing SARS-CoV-2 research. Considering the critical role of the RBD in mediating viral entry; the tendency of the RBD of SARS coronaviruses to be highly variable (21); and the recent emergence of new variants B.1.1.7, P.1, and B.1.351 (6–8, 22); increasing interest has been directed toward the surveillance of SARS-CoV-2 RBD mutations. Of concern is the observation that several of these SARS-CoV-2 RBD mutations escape monoclonal antibody (mAb) neutralization and/or attenuate polyclonal plasma neutralization (23–27). Subsequently, there has been a more cautious shift in assessing the efficacy of current vaccine and antibody therapies with respect to the continuously emerging genetic variants, especially since some vaccine candidates and antibody therapies solely target the WT RBD (28, 29).

At present, there is no standardized method for assessing the antibody-mediated neutralization of SARS-CoV-2, which can account for variation between studies (30). Assays that evaluate the ability of NAbs to inhibit viral replication in target cells by observing plaque reduction or a cytopathogenic effect are currently regarded as benchmark assays. The pseudovirus-based neutralization assays (31), which reduce the biosafety requirements when working with SARS-CoV-2, can also be used; however, these cell culture–based assays can be challenging to implement and time-consuming to run, which limits scalability. Until recently these were the only conventional assays available for evaluating NAbs. Limitations of the aforementioned assays have, however, motivated efforts to develop alternative assays, such as the surrogate virus neutralization test (32), which mirrors the RBD-ACE2 interaction in an ELISA plate format; a novel fluorescence reporter cell-based assay (33); and lentiviral particle fluorescent neutralization assays (23, 34). These assays are, however, limited to screening NAbs directed against only 1 RBD mutant at a time. In addition, these assays allow antibodies to incubate and neutralize SARS-CoV-2 before incubation with soluble ACE2 or ACE2-expressing cells. This approach may bias toward maximal NAb neutralization in comparison with a competitive approach. Therefore different approaches may need to be considered, depending on the context of antibody-mediated neutralization in vivo, e.g., vaccination versus passive monoclonal antibody therapy. The absence of a competitive assay to evaluate the neutralizing potential of SARS-CoV-2 antibodies against multiple emerging RBD variants simultaneously thus presents a major gap for SARS-CoV-2 surveillance and effective vaccine and antibody therapy development.

Herein we describe the development of a novel high-throughput RBD-ACE2 multiplex inhibition assay that measures SARS-CoV-2 NAbs against multiple RBD natural variants simultaneously. Bead-based multiplex assays allow for the assessment of multiple antigen-coupled beads in a single well. This assay format provides several key advantages, including less sample volume being required, reduced user operator time, and the ability to reliably detect analytes across a broad dynamic range, providing a much higher resolution of data than conventional immunoassays, such as ELISA (35, 36). We describe the validation of this rapid high-throughput multiplex assay against the cell-based microneutralization assay and evaluate how a selection of naturally occurring single–amino acid RBD variants observed during viral surveillance affect the potential efficacy of mAb therapeutics and the recognition by polyclonal convalescent human plasma.

## Results

**Figure 1:**
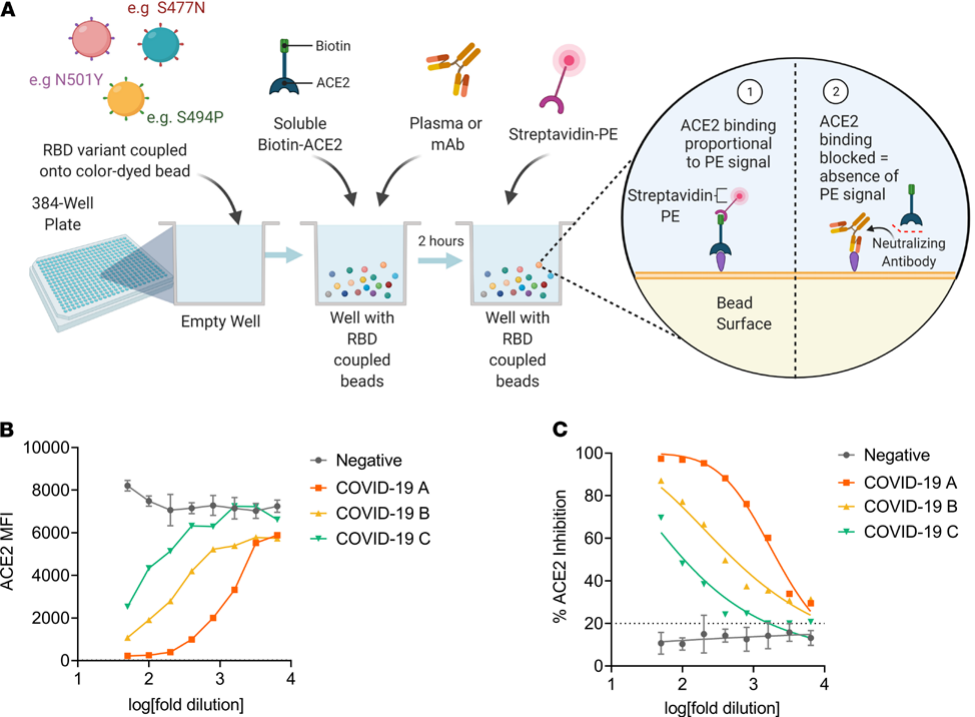
Principle of the SARS-CoV-2 inhibition multiplex assay. (**A**) Schematic representation of the RBD multiplex assay: anti–SARS-CoV-2 RBD antibodies compete with biotin-conjugated ACE2 for binding to RBD variants coupled to magnetic microspheres. Illustration was created using BioRender. (**B**) Inhibition of the RBD-ACE2 interaction by patients recovered from SARS-CoV-2 infection (COVID-19 patients A, B, and C) versus mean ± SD of (*n* = 7) COVID-19–negative samples, with a decrease in MFI observed as anti–SARS-CoV-2 neutralizing antibodies present in the sample blocked the ACE2–binding RBD coupled to magnetic microspheres. (**C**) Inhibition of the SARS-CoV-2 RBD–ACE2 interaction by plasma from convalescent COVID-19 patients, and mean ± SD of (*n* = 7) COVID-19–negative samples, with the determined cutoff at 20% inhibition indicated by the dotted line. Illness severity was severe for example COVID-19 patient A (orange squares), moderate for B (yellow triangles) and mild for C (green inverted triangles).

With the development of alternative ELISA-based assays, which mimic the virus–receptor interaction (32, 37), we sought to evaluate whether a similar approach could be adapted to a high-throughput manner. Here, we used a bead-based multiplex assay to evaluate the neutralizing capacity of SARS-CoV-2 antibodies against recombinant RBD variants. This was achieved by coupling each individual RBD variant to a distinct bead region with a defined distinctive spectral signature. As such, multiple RBD-coupled beads can be assayed simultaneously within the same well. Convalescent plasma or purified mAbs can then be allowed to simultaneously compete with soluble biotin-ACE2 for binding to the RBD variants coupled to the magnetic beads (Figure 1A). As a result, the neutralizing capacity of a sample against multiple RBD variants within each well is measured. This approach was first validated using only WT (Wuhan strain) RBD of SARS-CoV-2 to demonstrate proof of concept, before subsequent development of a multiplexed assay containing 24 SARS-CoV-2 natural RBD variants.

### Validation of a multiplex RBD-ACE2 inhibition assay to measure SARS-CoV-2 NAbs in plasma against WT RBD.

**Figure 2:**
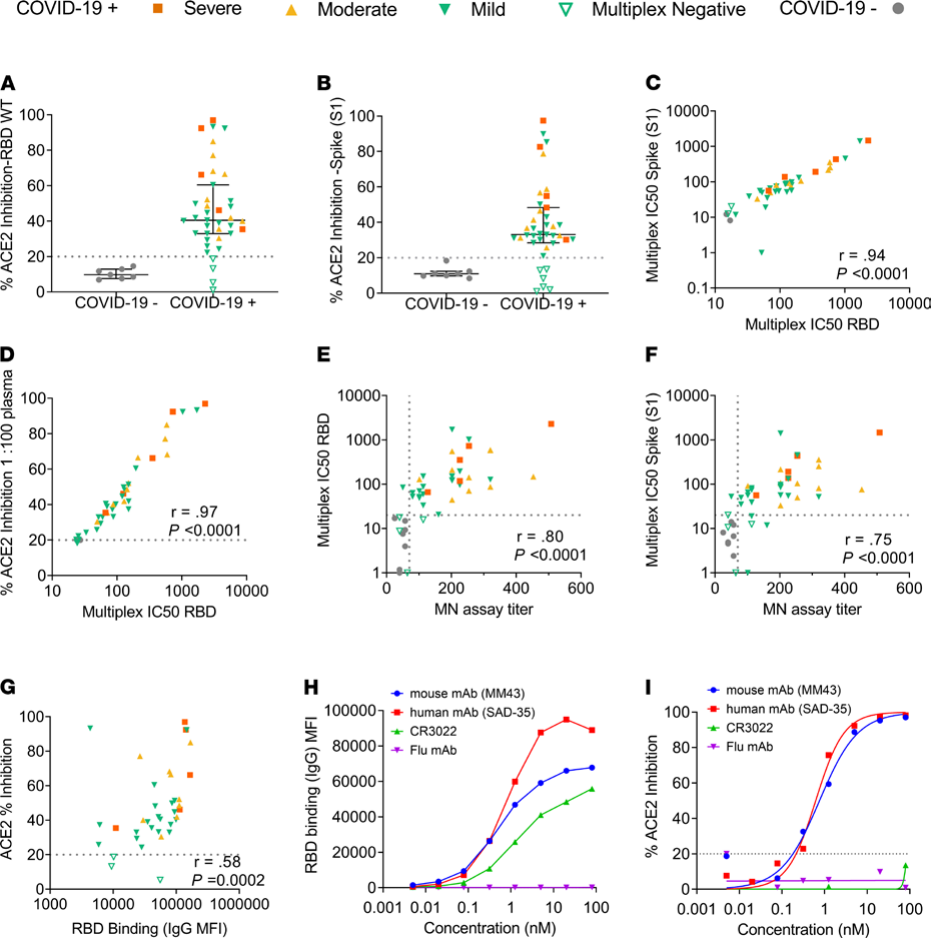
Validation of the SARS-CoV-2 inhibition multiplex assay. (**A**) Plasma samples from patients recovered from SARS-CoV-2 infection (*n* = 39) and SARS-CoV-2–negative healthy controls (*n* = 7) were tested for inhibition of the RBD-ACE2 interaction and (**B**) the S1-ACE2 interaction. SARS-CoV-2–positive subjects are colored based on COVID-19 illness severity. Orange squares, severe; yellow triangles, moderate; green inverted triangles, mild; gray, SARS-CoV-2–negative controls. COVID-19 subjects who tested negative on the multiplex assay are indicated by hollow symbols. (**C**) Spearman’s correlation between relative S1 and RBD IC_50_ percentage inhibition values. (**D**) Spearman’s correlation of the percentage ACE2 inhibition at a 1 in 100 dilution of plasma to RBD and the relative IC_50_ value. (**E**) Spearman’s correlation of multiplex ACE-RBD inhibition assay IC_50_ values and the titer obtained with the virus microneutralization assay. (**F**) Spearman’s correlation of multiplex ACE-Spike (S1) inhibition assay IC_50_ values and the titer obtained with the virus microneutralization assay. (**G**) Spearman’s correlation of ACE-RBD inhibition and (**H**) RBD binding of control mAbs. (**I**) Percentage ACE2 inhibition of control mAbs. Cutoff values to establish the positive/negative thresholds are indicated by dotted lines.

A subset of previously described (38) plasma samples collected from a cross-sectional cohort of Australian adults recovered from SARS-CoV-2 infection was chosen to first evaluate the performance of this assay with regard to its ability to quantitate ACE2-inhibitory antibodies from SARS-CoV-2 convalescent subjects. Subsequently, we then sought to determine whether NAbs in the SARS-CoV-2–positive plasma could inhibit ACE2 receptor binding in a similar manner as the virus microneutralization assay (39). In this assay, linearity was observed as inhibition of the SARS-CoV-2 RBD-ACE2 interaction by antibodies in SARS-CoV-2 convalescent plasma as a dose-dependent decrease in fluorescent ACE2 measured as MFI bound to immobilized RBD, whereas the MFI of SARS-CoV-2–negative plasma remained high across the 8-point 2-fold serial dilution (Figure 1B), demonstrating that the specific inhibition of the SARS-CoV-2 RBD-ACE2 interaction by plasma from convalescent SARS-CoV-2 patients occurs in a dose-dependent manner (Figure 1C). The capacity of antibodies in SARS-CoV-2 convalescent plasma to inhibit the interaction between ACE2 and RBD in this assay was detected in most patients in this cohort (median, 40.5%; IQR, 32.9%–60.4%), with 31% of patients exhibiting more than 50% ACE2 inhibition activity (Figure 2A). In comparison, slightly less than a quarter (23%) of patients exhibited more than 50% inhibition activity against S1 (median, 33.1%; IQR, 28.4%–48.3%; Figure 2B), suggesting that the full S1 protein may provide additional stability to the ACE2-RBD interaction. A nominal cutoff of 20% was set based on a panel of SARS-CoV-2–negative patients (Supplemental Figure 1A; supplemental material available online with this article; https://doi.org/10.1172/jci.insight.150012DS1). To further validate this assay, each plasma sample’s relative 50% inhibitory concentration (IC_50_) titer to RBD (WT) and spike (S1) was determined from the 2-fold 8-point dilution. Relative IC_50_ values of the ACE2-RBD versus ACE2-S1 interaction by plasma NAbs strongly correlated (*r* = 0.94, *P* *<* 0.0001; Figure 2C), and calculated ACE2 percentage inhibition at a 1:100 dilution of plasma positively correlated with RBD IC_50_ (*r* = 0.97, *P* < 0.0001; Figure 2D), suggesting that a 1:100 dilution of plasma would be closely representative of the relative IC_50_.

In order to determine the accuracy of this assay, that is, how well it is able to detect NAbs in comparison with a virus neutralization assay using SARS-CoV-2 infection of Vero cells (39), relative IC_50_ values to RBD obtained via the multiplex assay were correlated to the benchmark cell-based assay. Furthermore, since the multiplex assay is intended as a rapid surrogate assay for functional cell-based serological methods, spike (S1) responses were also correlated to the microneutralization assay for comparison. As shown in Figure 2, E and F, there was a strong correlation between the 2 assays for both RBD-ACE2 inhibition (*r* = 0.8, *P* < 0.0001) and S1-ACE2 inhibition (*r* = 0.75, *P* < 0.0001).

In addition, we examined the relationship between ACE2 inhibition and IgG RBD binding (Figure 2G) and found a moderate correlation between the two (*r* = 0.58, *P* = 0.0002), which suggests that inhibition may also be mediated by other antibody isotypes, such as IgA and IgM (40). Upon validation of the ACE2-RBD multiplex inhibition assay (Supplemental Table 2), we detected inhibition of ACE2 binding activity in 34 out of 35 COVID-19–positive samples that showed SARS-CoV-2 neutralizing activity in the microneutralization assay, resulting in a sensitivity of 97.14%. We identified 1 COVID-19 patient sample that tested negative in the microneutralization assay but tested positive in the multiplex assay, resulting in a specificity of 90.9%. All COVID-19–negative patients tested negative in both the RBD multiplex inhibition assay and the cell-based microneutralization assay. The multiplex assay was determined to be robust (*r*^2^ = 0.9; Supplemental Figure 1B) by comparing the repeatability of the percentage ACE2-RBD inhibition values obtained from the cohort of plasma samples tested by 2 operators in 2 independent experiments on separate days, with the mean percentage ACE2-RBD inhibition once again demonstrated to correlate strongly to the microneutralization assay (Supplemental Figure 1C).

Considering mAbs are of increasing interest for therapy or prophylaxis of SARS-CoV-2, we evaluated the ability of this assay to specifically measure mAbs that neutralize the ACE2-RBD interaction. Here we tested 2 commercially available neutralizing antibodies (1 human and 1 mouse) and 2 negative controls for RBD binding and ACE2 inhibition. The first negative control was an unrelated human mAb against influenza, and the second was CR3022, a mAb that has been described to bind RBD at an epitope that does not overlap with the ACE2 binding site of SARS-CoV-2 and therefore is unable to block the ACE2-RBD interaction (41). As demonstrated in Figure 2, H and I, both the human and mouse neutralizing mAbs demonstrated a very similar pattern of RBD binding and ACE2 inhibition. In contrast, the absence of binding and inhibition of ACE2 by a nonspecific influenza mAb confirmed the specificity of this assay. CR3022 binding to RBD coincided with the absence of ACE2 inhibition, further validating the specific ability of this assay to evaluate NAbs that specifically inhibit the RBD-ACE2 receptor interaction.

### Validation of an RBD natural variant multiplex assay.

**Figure 3:**
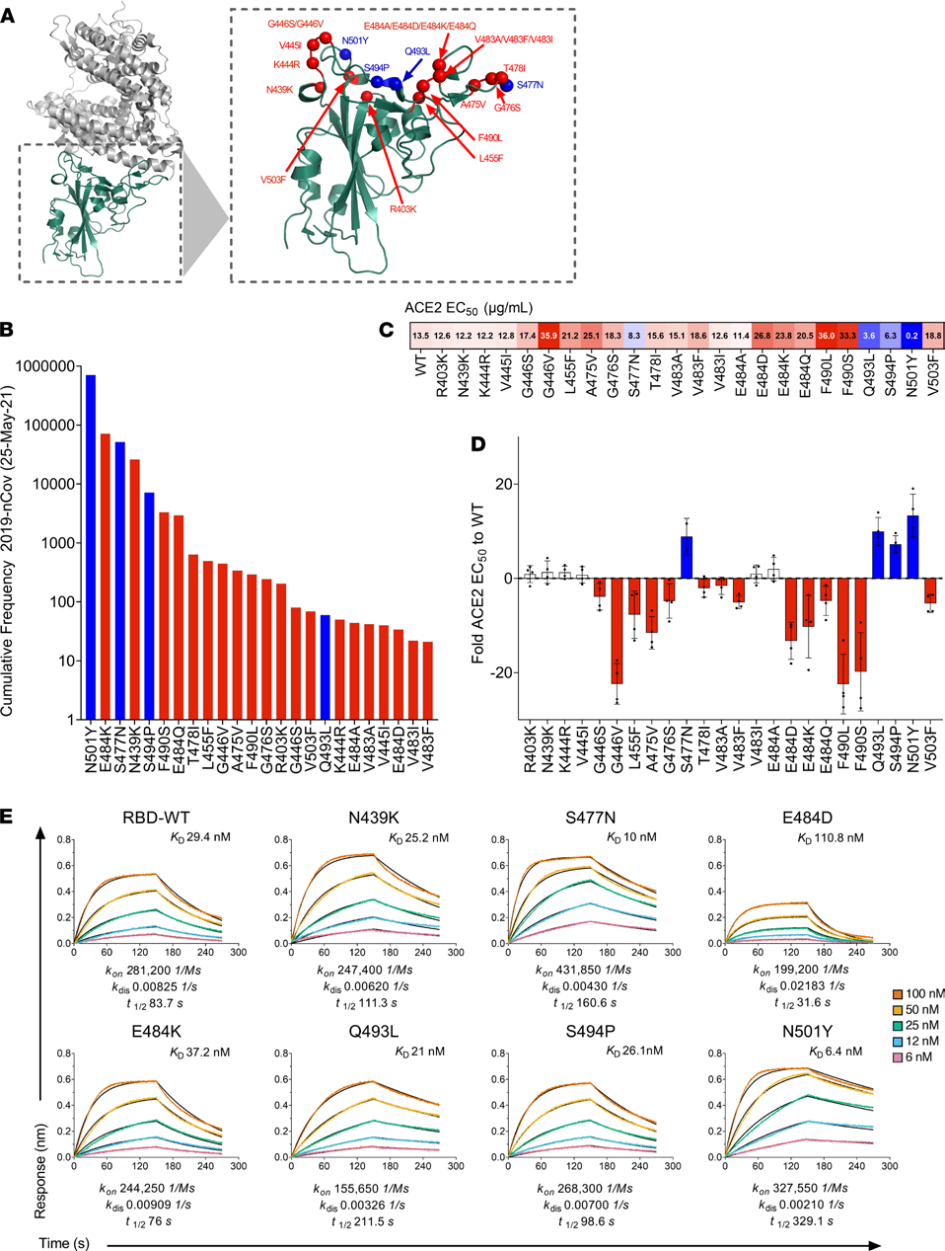
Characterization of RBD variants in the multiplex array. (**A**) Structure of novel coronavirus spike RBD complexed with its receptor, ACE2 (PDB ID code 6LZG), highlighting the amino acid positions of 24 RBD natural variants selected from the GISAID repository in June 2020, with the subsequent inclusion of S477N, and their proximity to the RBD-ACE2 interface. The structural illustration was generated using PyMol. Variants with a significantly higher affinity to ACE2 as determined in our multiplex assay are highlighted in blue. (**B**) Cumulative frequency of variants ranked from most frequently isolated to less frequent included in the multiplex array. Frequency of genomes is based on high-quality 2019-nCov genome sequences reported by the GISAID database as of May 25, 2021. Variants with a significantly higher affinity to ACE2 as determined in our multiplex assay are highlighted in blue. (**C**) Relative EC_50_ values (μg/mL) obtained for each of the variants. Values are color-coded blue for EC_50_ less than 10 μg/mL and red for EC_50_ values more than 10 μg/mL. (**D**) Mean fold difference for each RBD variant EC_50_ value relative to WT RBD. Data are presented as dot plots of (*n* = 4) independent experiments, with the mean ± SEM. Bars for variants with more than a 2-fold difference in affinity to the WT RBD are colored blue. Bars for variants with a slightly increased affinity (< 2-fold) are colored white. Variants with a weaker EC_50_ value relative to WT are colored red. (**E**) Bio-layer interferometry sensograms of immobilized ACE2 with 2-fold 6–100 nM serial dilutions of SARS-CoV-2 RBD variants in solution. Binding curves representative of 2 independent experiments for each variant are plotted (solid-colored lines), globally fitted to a 1:1 binding model (black line).

Upon validation of the multiplex RBD-ACE2 assay with WT RBD, we sought to expand the assay by multiplexing 24 RBD natural mutants selected from the GISAID RBD surveillance repository at the time (June 2020). This included the RBD variant S477N, which emerged and rose to be the second most frequent variant in the following months. A total of 25 variants, including the WT, were studied. Figure 3A illustrates the position of these variants on the RBD, and Figure 3B displays the more recently observed frequency of these variants according to the GISAID repository, with variant N501Y, currently the most frequent RBD variant worldwide (a key mutation present in the newly emergent B.1.1.7, B.1.351, B.1.1.70, and P.1 strains). The RBD variant assay was set up by coupling each of these 25 variants to an individual “bead region” corresponding to a unique spectral signature, with coupling efficiency across the RBD variant proteins onto the carboxylated magnetic microspheres verified with an anti-His Tag antibody (Supplemental Figure 2). This was determined by comparing the MFI of each of the coupled RBD proteins to the WT, with coupling across all the RBD variants determined to be comparable to the WT RBD. We also correlated the IgG binding (*r*^2^ = 0.97) and ACE2 inhibition (*r*^2^ = 0.98) of each variant on its own (single plex) as well as when in combination with all other variants (multiplexed) (Supplemental Figure 3, A and C). Furthermore, MFI obtained between single plex and multiplex was also compared via a paired *t* test (Supplemental Figure 3, B and D). Differences were not significant, which along with the correlation analysis confirmed the absence of any undesirable effect or significant loss of signal as a result of multiplexing all RBD variant proteins simultaneously in a single well.

### Affinity of RBD variants to ACE2.

To evaluate how the cocktail of RBD variants affected binding to ACE2 in our assay, a 2-fold 12-point serial dilution of ACE2 starting from a final concentration of 160 μg/mL per well was performed, and a relative EC_50_ for each of the variants was determined (Figure 3C). The calculated difference in the relative EC_50_ of each variant was then compared to RBD WT (Figure 3D), with the majority of RBD variants exhibiting a similar or weaker relative EC_50_ when compared to the WT RBD, a trend consistent with the observation that many missense mutations often negatively affect ACE2 binding (42). Nevertheless, we found some variants demonstrated significantly enhanced binding to ACE2 relative to WT SARS-CoV-2 RBD. These were N501Y, Q493L, S494P, and S477N, which demonstrated the lowest overall relative EC_50_ values (0.2, 3.6, 6.3, and 8.3 μg/mL, respectively) as determined by our ACE2-RBD multiplex assay (Figure 3C).

Though it is now well-known that N501Y, which is the shared mutation in the B.1.1.7, B.1.351, B.1.1.70, and P.1 lineages, is predicted to have a higher affinity for ACE2 (42), here we sought to characterize the binding kinetics of N501Y to WT RBD and other potential high-affinity variants. Based on the relative EC_50_ values observed in our multiplex assay, we selected these high -affinity variants, and the emerging N439K variant present in currently circulating lineage B.1.258, as well as E484K, which is a mutation shared by B.1.351, B.1.525, and P.1 lineages, to be profiled and compared for their binding kinetics to ACE2 using bio-layer interferometry (BLI). In addition, 1 variant that showed a reduced affinity for ACE2 (E484D) was selected to be profiled for comparison.

BLI profiles of the RBD variants binding to ACE2 (Figure 3E) showed that indeed variant N501Y demonstrated an enhanced affinity for the ACE2 receptor, with an almost 5-fold increase in affinity (*K_D_* 6.4 nM vs. 29.4 nM), which was driven by a much slower off rate (k_dis_) of this variant (*t_1/2_* 329.1 seconds [k_dis_ 0.00210 1/s] vs. *t_1/2_* 83.7 seconds [k_dis_ 0.00825 1/s]) compared with the WT. Variant S477N also demonstrated enhanced (*K_D_* 10 nM) affinity for ACE2, although variant Q483L (*K_D_* 21 nM) demonstrated a slower off rate, *t_1/2_* 211.5 seconds versus *t_1/2_* 160.6 seconds by comparison. N439K and S494P had weakly enhanced overall affinity for ACE2, *K_D_* 25.2 nM and 26.1 nM, respectively, compared to the WT RBD (29.4 nM). The enhanced affinity of N439K was the result of the almost 1.5-fold slower off rate, *t_1/2_* 111.3 s (k_dis_ 0.00620 1/s) versus *t_1/2_* 83.7 s (k_dis_ 0.00825 1/s) of the WT. Confirming multiplex ACE2 EC_50_ assay observations, variants E484K and E484D demonstrated reduced affinity to ACE2 (*K_D_* 37.2 nM and *K_D_* 110.8 nM, respectively).

### Mapping mutations to the SARS-CoV-2 RBD that affect recognition by mAbs.

**Figure 4:**
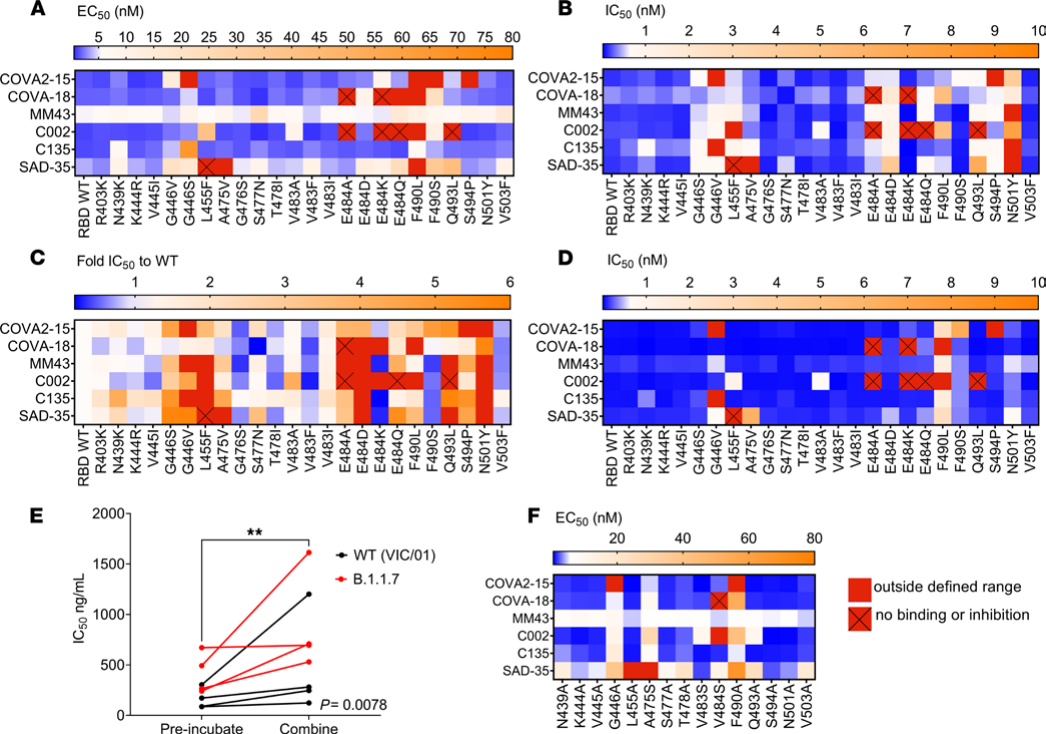
Mapping mutations to the RBD that affect recognition by mAbs. (**A**) Relative EC_50_ RBD binding of each mAb to each of the RBD variants. (**B**) Relative IC_50_ RBD inhibition of each mAb to each of the RBD variants. (**C**) RBD natural variant ACE2-RBD relative IC_50_ inhibition relative to RBD WT. Blue, stronger inhibition of RBD variants relative to WT (IC_50_ < RBD WT). Orange, weaker inhibition of RBD variants relative to WT (>1- to 6-fold RBD WT IC_50_). Red, more than 6-fold weaker RBD WT IC_50_, i.e., very poor/absence of inhibition. (**D**) Relative IC_50_ RBD inhibition of each mAb to each of the RBD variants performed in a noncompetitive format where mAbs were preincubated prior to addition of ACE2. (**E**) Wilcoxon’s paired 2-tailed *t* test comparison of IC_50_ values obtained via microneutralization assay for virus preincubated with mAb for 1 hour at 37°C (preincubated) versus addition of mAb, virus, and cells (combined). (**F**) Relative EC_50_ binding of mAbs to alanine (or serine if previously alanine) RBD mutants generated of selected RBD variants via multiplex. **P < 0.01.

We next sought to apply the multiplex assay to map potential escape mutations using a panel of 6 previously characterized mAbs. These were human mAbs COVA2-15 and COVA-18, (43), C002 and C135 (44), and SAD-35 (Acro Biosystems) and mouse mAb MM43 (Sino Biological). First, we assessed binding of each mAb to the RBD variants by determining the relative EC_50_ (Figure 4A), and second, we determined ACE2 inhibition of these mAbs to these variants by calculating the relative IC_50_ (Figure 4B), from an 8-point 4-fold serial dilution of each mAb. Though all 6 mAbs bound (Figure 4A) and inhibited (Figure 4B) the SARS-CoV-2 RBD WT with relatively high affinity, they differed in the extent to which they bound and inhibited each of the RBD variants. Variants with mutations at positions 446 and 484 (with the exception of E484D) of the RBD prominently demonstrated antibody escape and consequently poor inhibition. COVA2-15 and C135 demonstrated escape to G446V, while COVA2-15 demonstrated a loss of binding and inhibition to S494P. C002 demonstrated escape from variants E484A, E484K, E484Q, and Q493L. Furthermore, N501Y demonstrated an overall reduction in its ability to be inhibited across all mAbs as demonstrated by significantly weaker relative IC_50_ values, compared with the WT (Figure 4C), despite all mAbs having the capacity to bind RBD variant N501Y with relatively high affinity (Figure 4A).

We next generated a panel of 15 of the RBD variants where we mutated the amino acid variants to alanine (or to serine if alanine was already present; Figure 4F), in order to confirm which amino acid positions are essential for mAb epitope binding. These RBD alanine mutants were subsequently coupled to multiplex beads and incorporated into our multiplex assay. We found that for a subset of RBD variants, immune escape occurred due to a reduction or absence of mAb binding at key amino acids, such as positions 446 and 490. In contrast, other positions, such as 493, 494, and 501, were relatively unaffected by the alanine substitution, suggesting that alternative immune escape mechanisms beyond loss of mAb recognition may be involved.

The majority of current in vitro SARS-CoV-2 neutralization assays first allow antibodies to bind the RBD before subsequent incubation with the ACE2 receptor or ACE2-expressing cells. This approach has the potential to bias the assay to allow for maximal NAb neutralization, thus reducing the influence of RBD variant affinity to the ACE2 receptor. In light of this, we sought to evaluate how mAb preincubation followed by incubation with the ACE2 receptor compared with our original competitive format of the ACE2-RBD inhibition assay, where all components were added simultaneously. Preincubation of mAbs in the absence of ACE2 was found to significantly overestimate the neutralizing capacity of the mAbs (Figure 4D), conferring the mAb a competitive advantage by allowing it to neutralize the RBD in the absence of competing ACE2. Although similarities in the overall trend of neutralization compared to the WT emerged (Supplemental Figure 4A), the much lower relative IC_50_ values obtained in comparison with the competitive assay performed in parallel (Figure 4D) appreciably reduced the resolution of the differences in the neutralizing response between variants, in particular to N501Y, to which all mAbs demonstrated an attenuated neutralizing response in the competitive assay (Figure 4B). This observation was, however, not as apparent in the noncompetitive assay (Figure 4D). The sequence of reagent addition was also tested in a cell-based live virus microneutralization assay (Figure 4E), with preincubation of WT (VIC/01) or B.1.1.7 (RBD N501Y variant) virus and antibody compared to a competitive (combined) approach where virus and mAb were added together into the plate with ACE2-expressing cells. Though no differences between the WT (VIC/01) or B.1.1.7 virus were observed in this case (Supplemental Figure 5), a notable difference (*P* = 0.0078) between the 2 approaches was observed with preincubation of virus with antibody biasing toward maximal neutralization (Figure 4E).

Cocktails of 2 or more mAbs may provide greater coverage of breadth of protection across multiple SARS-CoV-2 RBD variants. Thus, correlations of the ACE2-RBD IC_50_ inhibition were performed between the different mAbs to identify which combinations of mAbs were most similar in breadth of inhibition (Supplemental Figure 4B, blue combinations) and, importantly, which combinations of mAbs had the capacity to inhibit the most diverse range of RBD variants (Supplemental Figure 4B, pink/red combinations), informing selection of complementary mAb candidates. From the panel of mAbs tested, COVA2-15 had the most distinct RBD variant inhibition in comparison with other mAbs, suggesting that it would complement the RBD variant inhibition provided by the other mAbs.

### Mapping mutations to the SARS-CoV-2 RBD that affect recognition by convalescent human plasma antibodies.

Having established the multiplex of RBD natural variants and tested these with a panel of mAbs, we sought to evaluate recognition of the variants by polyclonal immune plasma from 20 individuals who had recovered from SARS-CoV-2 infection a mean of 36 days earlier (Supplemental Table 1). These samples were collected early in the pandemic (March–April 2020), before the detection of the N501Y variant in Australia.

**Figure 5:**
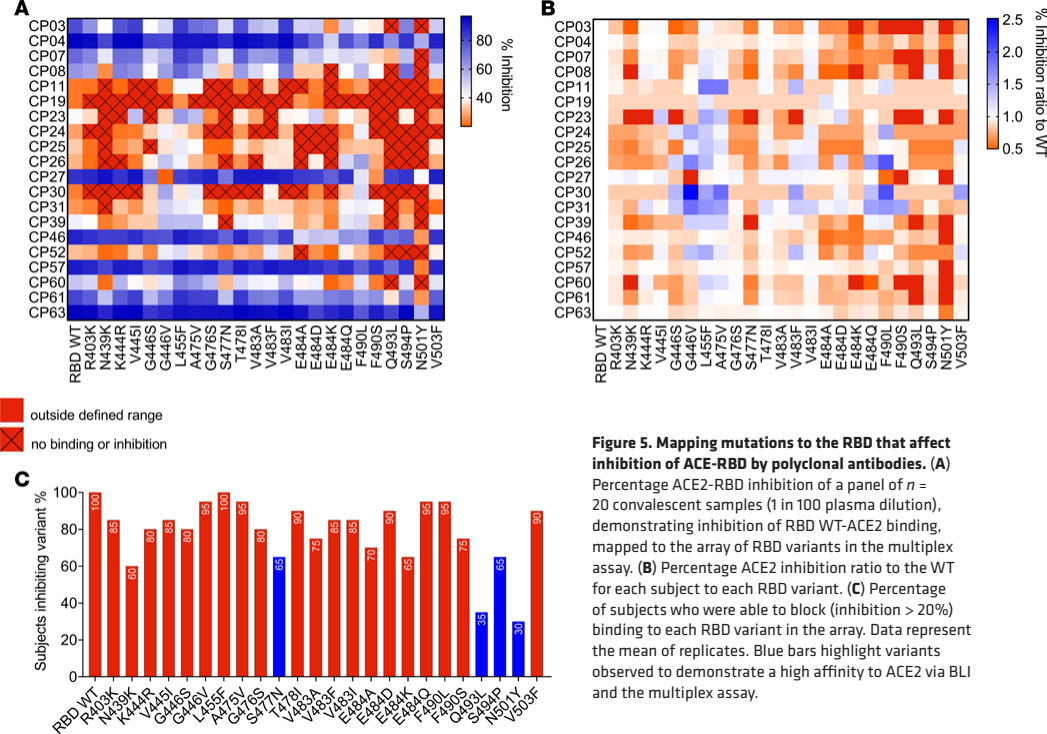
Mapping mutations to the RBD that affect inhibition of ACE-RBD by polyclonal antibodies. (**A**) Percentage ACE2-RBD inhibition of a panel of *n* = 20 convalescent samples (1 in 100 plasma dilution), demonstrating inhibition of RBD WT-ACE2 binding, mapped to the array of RBD variants in the multiplex assay. (**B**) Percentage ACE2 inhibition ratio to the WT for each subject to each RBD variant. (**C**) Percentage of subjects who were able to block (inhibition > 20%) binding to each RBD variant in the array. Data represent the mean of replicates. Blue bars highlight variants observed to demonstrate a high affinity to ACE2 via BLI and the multiplex assay.

Plasma samples were tested at a single-point dilution of 1 in 100 across the natural RBD variants, based on the strong correlation of this dilution with relative IC_50_ values (Figure 2D). As shown in Figure 5A, most subjects demonstrated a reduction in the inhibition of most RBD variants, with the neutralizing response to variant N501Y being the most significantly attenuated, with many subjects demonstrating a ratio of less than 0.5 to that observed with the WT (Figure 5B), equating to over a 2-fold decrease in percentage inhibition to variant N501Y compared with the WT. Similar to the findings observed with the panel of mAbs, many convalescent plasma samples were able to bind the N501Y mutant with similar affinity to WT (Supplemental Figure 6). However, likely due to the enhanced affinity of N501Y to ACE2, polyclonal convalescent plasma demonstrated a reduced ability to inhibit the ACE2-RBD interaction (Figure 5, A and B) to this variant. Figure 5C illustrates the percentage of patients who had a neutralizing response (i.e., >20%) to each RBD variant, with the lowest proportion of responders observed to occur to high-affinity variants N501Y (30%) and Q493L (35%). This was followed by N439K (60%), N477N (65%), and E484K (65%). Though antibody neutralization is a complex phenomenon, and true protection potential in vivo is difficult to predict, our findings suggest that despite having the capacity to inhibit WT RBD, it is possible that some convalescent individuals may not generate antibodies with high enough affinity to compete with RBD variants with an enhanced affinity to ACE2.

## Discussion

To date, numerous SARS-CoV-2 RBD variant viruses have emerged, with the number and frequency of these variants steadily increasing since the beginning of the pandemic. Of further concern has been the observation that a number of these variants demonstrate escape from antibody neutralization (23–27, 45), with the more recent emergence of new variants with multiple mutations in the RBD (8) also demonstrating resistance to in vitro antibody neutralization (46). In the context of increasing population-level immunity to WT SARS-CoV-2, and the rollout of vaccines and antibody therapies targeting the RBD, mapping the antibody response to emerging RBD mutations will be critical to guide future preparedness efforts by enabling preemptive forecasting of mutations that might affect antibody recognition.

To address the need to rapidly survey the neutralizing capacity of antibodies against RBD variants, here we describe a rapid high-throughput multiplex assay to measure RBD binding and NAbs to 24 natural RBD variants simultaneously. Using a panel of plasma samples obtained from subjects previously infected with SARS-CoV-2, we found this assay correlated well with a cytopathic effect virus microneutralization assay. Though this assay is not intended to replace gold standard SARS-CoV-2 cell-based neutralization assays, it provides a simple solution for broadly surveying the diversity of SARS-CoV-2 neutralizing specificities to multiple RBD mutants in a rapid high-throughput format, which can easily adapt to include new RBD variants as they emerge. Furthermore, this assay presents several key advantages over existing assays, since there is no need for viruses, cells, biosafety containment requirements, or highly skilled tissue culture operators. Finally, results can be obtained rapidly on the same day in a high-throughput manner with the use of minimal sample volumes.

It has now been reported by several studies (23–27, 45) that antibody neutralization against some RBD mutants is reduced, suggesting that SARS-CoV-2 has mutated to evade host immunity. Profiling of a subset of previously published mAbs and polyclonal convalescent plasma samples, we demonstrate the heterogeneity of the neutralizing response to a multiplex of RBD variants. Specifically, within the mAb panel, we found variants at positions 446 and 484 (with the exception of E484D) were poorly neutralized. We also found variant G446V, which has demonstrated resistance to REGEN10987 (46) and C135 (27), to be poorly neutralized by our panel of mAbs. Furthermore, F490L, which has been described to be remarkably resistant to mAbs (45), demonstrated weak binding to the panel of mAbs in comparison with the other variants in the multiplex assay, with RBD alanine mutation at this position confirming that this is a critical epitope for certain mAbs. Despite this, we found most mAbs were able to demonstrate a modest inhibition at position 490. It is important to note that residues L455, A475, F490, and Q493 are residues that have been previously reported to be SARS-CoV-2 RBD-ACE2–interacting residues based on structure (12, 47). Unlike the previously described variants, which demonstrated escape from mAb recognition, N501Y was recognized at relatively high affinity by most mAbs. However, inhibition was significantly attenuated across some mAbs in our assay (Figure 4B). Similar findings to N501Y pseudotyped and chimeric viruses have been reported for some mAbs (48, 49), with partial escape from COVA1-18 and COVA2-15 associated with the N501Y mutation (48). Our findings for mAb C135 and C002 contrast those of others, which demonstrated little to no significant impact of the N501Y variant (27, 50).

When we profiled the polyclonal response of SARS-CoV-2 convalescent plasma to the RBD variants by multiplex, we observed a similar pattern in the attenuation of the inhibitory response to variants at position E484, as well as a pronounced reduction in the inhibitory response against N501Y (as well as Q493L, followed by N439K and S477N). Though the E484K variant has been associated with immune escape because it is situated in an immunodominant epitope targeted by most infected patients (23–27, 45), several studies have found minimal to no pronounced effect of the N501Y mutation on the neutralizing activity of plasma from convalescent or vaccinated individuals (51–53). On the other hand, other recent studies have found a modest approximately 2- to 3-fold drop for plasma from convalescent and vaccinated individuals (48, 54–56). A proportion of RBD-specific mAbs isolated from vaccinated individuals have also demonstrated significant neutralization loss (>50-fold) against N501Y mutants (57). It has also been suggested that mutations of the RBD, such as N501Y, that increase the affinity of ACE2 are likely to tip the equilibrium away from mAb-RBD interaction toward RBD-ACE2, making the virus more difficult to neutralize (54). It is, however, generally thought that minimal or modest drops in neutralization do not indicate a biologically relevant change in neutralization activity, and there is presently no evidence for vaccine escape from the B.1.1.7 variant carrying the single RBD N501Y mutation (58). The unique format and competitive approach of our assay, which specifically examines responses to the RBD removed from the context of the spike trimer, thus make our findings difficult to align with other studies in the literature. It is therefore plausible that factors in the assay design, including the orientation by which RBD may be being presented on the beads and the use of the RBD instead of the spike, may exaggerate the impact of the N501Y mutation due to its altered affinity.

The present study also observed improved neutralization in our multiplex and cell-based assay when antibody was preincubated first, in the absence of the ACE2 receptor (Figure 4, D and E). These findings suggest that a competitive format where both mAbs and ACE2 (or ACE2-expressing cells) are added, simultaneously, may allow for a more relevant assessment of the neutralizing capacity of NAbs, though in vivo relevance remains to be determined and is complicated by the context in which neutralization of SARS-CoV-2 takes place. The relationship of antibody to non–cell-bound virus in a vaccinated or convalescent individual, compared with treatment in an unvaccinated individual, or postexposure prophylaxis, thus would need to be taken into consideration.

We observed via BLI an almost 5-fold greater affinity of the N501Y variant to ACE2 compared with WT, confirming previous studies that describe the formation of 3 additional bonds that increase the affinity of this variant to the ACE2 receptor (22, 42). The EC_50_ of the different RBD variants to ACE2 as measured by the multiplex assay largely correlated to the affinity observed via BLI (Figure 3, D and E). However, there were differences in particular with S477N, which had the second highest overall affinity (*K_D_*) for ACE2 (after N501Y) as determined via BLI but had weaker EC_50_ compared with Q493L and S494P, respectively. This could be explained by the fact that compared with Q493L and S494P (which have a comparable *K_A_* to the WT), S477N has a much faster on rate, which results in a stronger overall *K_D_*. This would likely not be captured in the multiplex assay, which like an ELISA is more a reflection of the dissociation phase, i.e., what remains bound after wash steps. In addition, the multiplex assay is calculated based on the half-maximal response binding of soluble ACE2 to the RBD variant, whereas BLI kinetics are calculated on a globally fitted concentration series of soluble RBD binding to immobilized ACE2, which may also account for differences in affinity obtained by the 2 methodologies.

Although the E484K mutation has been associated with immune escape (27), predictions regarding its affinity to the ACE2 receptor are conflicting (22, 59). Our findings, which demonstrate a reduced affinity of E484K to ACE2, however, support the recent observations in which the E484K mutation has been found to prevent the formation of 2 salt bridges that help to form and stabilize the RBD-ACE2 complex, thus reducing ACE2 binding affinity (22). We also found S477N (60), and Q493L, a variant at a position that interfaces directly with ACE2, had enhanced affinity to ACE2, confirming observations from deep mutational scanning analysis (42). What these differences in affinity to the ACE2 receptor mean for the overall viral fitness and transmissibility of these variants is beginning to be explored (26). However, lessons learned from the original SARS-CoV outbreak, which have served to guide SARS-CoV-2 research, suggest that the infectivity of different SARS-CoV strains in host cells is correlated to the binding affinity between the RBD of each strain and ACE2 (61–63). Indeed, the frequency and presence of the N501Y mutation in recently rapidly emerging SARS-CoV-2 lineages, B.1.1.7, B.1.351, B.1.1.70, and P.1, appear to further strengthen this hypothesis, as does the frequency of S477N and S494P, which we find bound ACE2 with an enhanced affinity (Figure 3, B and D). It is interesting to note, however, that unlike higher affinity N501Y and S477N variants, F490L and G446V demonstrated the weakest EC_50_ values in our ACE2 binding assay (Figure 3D), but were observed at relatively high frequencies (Figure 3B), supporting the hypothesis that modestly deleterious mutations may have the potential to rise in prevalence if they confer escape from selective pressures, such as the immune response (27, 64). In this multiplex assay, we compared single–amino acid RBD variants; however, future studies aim to expand the bead array to incorporate the multiple amino acid mutations present in the RBD of newly emerging lineages, as well as other variants that have come to rise in frequency.

As SARS-CoV-2 continues to infect people globally, and variants with multiple mutations in the spike protein emerge, there is a growing urgency to rapidly develop effective and therapeutic options especially for high-risk individuals who may not develop a robust immune response or those that may not be able to be vaccinated. This high-throughput multiplex assay has the potential to rapidly flag variants that may potentially demonstrate an enhanced affinity to ACE2. It also characterizes the RBD neutralizing response in a competitive format, which takes into account the relative affinities of each RBD variant to the ACE2 receptor. In summary, the application of this novel assay thus covers a major gap in surveying and anticipating patterns of antibody resistance to emerging SARS-CoV-2 RBD, a factor which is becoming increasingly important for guiding the development of effective SARS-CoV-2 therapeutic agents to curb the ongoing pandemic.

## Methods

### Experimental samples and subject details

Patients who had recovered from COVID-19 and healthy controls were recruited through contacts with the investigators and were invited to provide a blood sample as previously described (38). For all participants, whole blood was collected with sodium heparin anticoagulant, and plasma was collected and stored at −80°C until use. Supplemental Table 1 describes the study samples used in the RBD variant-ACE2 inhibition assay.

### Recombinant proteins

#### RBD proteins.

A total of 24 SARS-CoV-2 RBD variants (Supplemental Table 3) were selected from the GISAID RBD surveillance repository (June 2020). The WHU1 WT isolate (2), SARS-CoV-2 RBD sequence (GenBank: MN908947.3; amino acids 319–541; RVQP…CVNF), with the signal peptide (amino acids 1–14; MFVF…VSSQ) and a hexahistidine tag subcloned into pcDNA3.4 vectors by GenScript Corporation, was used as the original WT sequence. RBD variants were expressed in Expi293 HEK cells (Thermo Fisher Scientific) and maintained in suspension at 37°C and 8% CO_2_. Cells were transfected at a density of 3 × 10^6^ with 1 μg of plasmid DNA per 1 mL of culture and ExpiFectamine 293 reagent diluted in Opti-MEM (Thermo Fisher Scientific) following the manufacturer’s protocol. A total of 22 hours after transfection, ExpiFectamine 293 Transfection Enhancers 1 and 2 (Thermo Fisher Scientific) were added to transfected cells along with lupin peptone (Solabia Group). A total of 6 days after transfection, the supernatant was collected by centrifugation at 4500*g* for 15 minutes at 4°C, and 10 mM MgCl_2_ was added to improve binding to the column and filtered through a 0.22 μm filter. RBD variants were purified by loading the supernatant onto a 1 mL Ni Excel column (GE Healthcare, now Cytiva). Columns were equilibrated and washed using Dulbecco’s phosphate-buffered saline (DPBS). RBD variants were eluted using 300 mM imidazole and 100 mM NaCl DPBS buffer. A second purification step was performed by loading the eluate on an Superdex 75 Increase 10/300 pg gel filtration column (GE Healthcare). Protein concentration was determined by absorbance measurement at 280 nm, and purity was determined using SDS-PAGE.

For the alanine-scan RBD mutant multiplex assay, the alanine mutants were generated by GenScript Corporation. Where an alanine was naturally present, a serine substitution was used instead. Recombinant proteins were expressed from HEK293 cells (Thermo Fisher Scientific) and subsequently purified using Ni-NTA columns (GenScript). Protein concentration was determined by absorbance measurement at 280 nm and purity was determined using SDS-PAGE.

#### Control proteins.

SARS-CoV-2 Spike S1 (40591-V08H, Sino Biological) recombinant protein was also included as a positive control in the multiplex assay while Influenza Hemaglutinin (H1N1 A/Cal/07/2009, 11085-V08H, Sino Biological), which does not bind to ACE2, was included as a negative control.

#### ACE2.

DNA encoding the truncated human ACE2 ectodomain (residues 19–613) with a C-terminal AVI-tag and 6xHis-tag was synthesized (IDT) and subcloned into a pHLSec expression plasmid. Plasmid DNA was used for transient expression in Expi293F cells using ExpiFectamine 293 Transfection Kits (Thermo Fisher Scientific). Expression supernatant was harvested at day 6 and dialyzed into 10 mM Tris pH 8.0. ACE2 was subsequently purified using weak anion exchange (DEAE Sepharose, Cytiva), followed by size-exclusion chromatography (Superdex 200, Cytiva). Protein was then biotinylated enzymatically using BirA enzyme and further purified using strong anion exchange (MonoQ, Cytiva).

#### Monoclonal antibodies.

Human IgG1 Anti-SARS-CoV-2 RBD neutralizing antibody (SAD-S35, Acro Biosystems) and mouse IgG2b SARS-CoV-2 RBD neutralizing antibody (40592-MM43, Sino Biological) were used as commercially available positive controls. An in-house influenza mAb and human monoclonal CR3022, which binds RBD at an epitope that does not overlap with the ACE2 binding site of SARS-CoV-2 and therefore shows no competition with ACE2 (41), were used as negative controls. The heavy and light chain sequences of previously described mAbs COVA2-15 and COVA-18 (43), and C002 and C135 (44), were synthesized and subcloned into human IgG1 expression plasmids. These were expressed in Expi293 cells and purified using Protein A (Thermo Fisher Scientific) as previously described (43).

### RBD multiplex assay

To study the activity of RBD-specific antibodies to inhibit ACE2 binding, we developed a custom high-throughput bead-based RBD multiplex assay that can simultaneously assess antibody inhibition of multiple RBD variants at once, therefore preserving time and the amount of sample required.

#### Coupling of RBD proteins to magnetic multiplex beads.

RBD proteins described above (Supplemental Table 3) were covalently coupled onto magnetic multiplex beads as previously described (65). Briefly, each respective RBD protein was coupled to a distinct magnetic carboxylated bead region (Bio-Rad), using a 2-step carbodiimide reaction at a ratio of 1 million beads to 10 μg of each RBD protein. Recombinant influenza hemagglutinin from H1N1 (A/Cali/07/2009) (11085-V08H, Sino Biological) and SARS-CoV-2 Spike S1 (40591-V08H, Sino Biological) were also coupled using identical protocols as control proteins. Coupling efficiency of the recombinant RBD was assessed using an anti-His Tag antibody (A00174, GenScript Corporation). A complete list of the magnetic bead regions and respective RBD variants is in Supplemental Table 3.

#### Competitive RBD variant–ACE2 multiplex inhibition assay.

The RBD-ACE2 multiplex inhibition assay was performed as follows: first, RBD variant–coupled beads and control protein beads were pooled together to form a bead cocktail (henceforth referred to as the bead cocktail). RBD multiplex assays were conducted in black, clear-bottom, 384-well plates (Greiner Bio-One). A total of 20 μL of the bead cocktail (which contained 700 beads of each bead region per well) was added to each well, along with 10 μL of diluted plasma or mAb at a starting final concentration of 80 nM per well, prepared as 8-point 4-fold titrations in assay buffer (0.1% BSA/PBS). A total of 20 μL of 25 μg/mL of Avi-tagged biotinylated ACE2 was subsequently added to all wells, with the exception of no-ACE2 control wells, in which 20 μL of assay buffer was added instead, bringing the total final volume per well to 50 μL. Each plate was incubated for 2 hours on a plate shaker at room temperature, before being washed twice in 0.05% PBS–Tween 20. ACE2 binding was detected with 40 μL of Streptavidin, R-Phycoerythrin Conjugate (S866, Thermo Fisher Scientific), at 4 μg/mL added for 1 hour, followed by the addition of 10 μL of 10 μg/mL of R-Phycoerythrin Biotin-XX Conjugate (P811, Thermo Fisher Scientific), then incubating for an additional hour. Plates were incubated on a plate shaker at room temperature before being washed 3 times. A total of 60 μL of sheath buffer was added to each well, with plates left to shake for 10 minutes on a plate shaker prior to acquisition on a FLEXMAP 3D (Luminex Corporation) following manufacturer’s instructions, acquiring a minimum of 50 beads per bead region. The binding of ACE2 was detected as phycoerythrin-labeled reporter, measured as MFI. A summary of reagents, concentrations, and volumes used per well is described in Supplemental Table 4.

The noncompetitive ACE2 inhibition assay was performed as above with the exception that antibody was allowed to incubate first for 1 hour with the RBD variant bead cocktail, before addition of biotinylated ACE2 for a further hour.

#### IgG binding to RBD variant multiplex assay.

RBD variant bead cocktails were added to each well of black, clear-bottom, 384-well plates as described for the RBD variant–ACE2 inhibition assay. A total of 10 μL of diluted plasma or mAb at a starting final concentration of 80 nM per well, prepared as 8-point 4-fold titrations in assay buffer (0.1% BSA/PBS), was subsequently added. Each plate was incubated for 2 hours on a plate shaker at room temperature, before being washed twice in 0.05% PBS–Tween 20. Relative RBD antibody binding was detected using anti-human IgG R-Phycoerythrin Conjugate (9040-09, Southern Biotech) at 1.3 μg/mL for 2 hours. Plates were incubated on a plate shaker at room temperature before being washed 3 times. A total of 60 μL of sheath buffer was added to each well, with plates left to shake for 10 minutes on a plate shaker prior to acquisition on a FLEXMAP 3D following manufacturer’s instructions, acquiring a minimum of 50 beads per bead region. The binding of IgG was detected as phycoerythrin-labeled reporter MFI.

### Microneutralization test

The virus microneutralization test was performed as previously described (38, 39). Briefly, SARS-CoV-2 isolate CoV/Australia/VIC01/2020 (66) was passaged in Vero cells, and samples were serially diluted before the addition of 100 TCID_50_ of SARS-CoV-2 in MEM/0.5% BSA and incubation at room temperature for 1 hour. Residual virus infectivity in the plasma/virus mixtures was assessed in quadruplicate wells of Vero cells incubated in serum-free media containing 1 μg/mL of trypsin treated with l-1-tosylamido-2-phenylethyl chloromethyl ketone (TPCK trypsin) at 37°C and 5% CO_2_; viral cytopathic effect was read on day 5.

### Microneutralization assay with ELISA-based readout

WT SARS-CoV-2 (CoV/Australia/VIC/01/2020) and B.1.1.7 CoV/Australia/VIC/179912 isolates were passaged in Vero cells (ATCC), and 96-well, flat-bottom plates were seeded with Vero cells (20,000 cells per well in 100 μL). The next day, Vero cells were washed once with 200 μL serum-free DMEM and added to 150 μL of infection media (serum-free DMEM with 1.33 μg/mL TPCK trypsin). Two-fold serial dilutions of mAbs (from 5 μg/mL) were incubated with WT and B.1.1.7 SARS-CoV-2 isolates at 2000 TCID_50_/mL at 37°C for 1 hour. Next, mAb-virus mixtures (50 μL) were added to Vero cells in duplicate and incubated at 37°C for 48 hours. “Cells only” and “virus+cells” controls were included to represent 0% and 100% infectivity, respectively. After 48 hours, all cell culture media were carefully removed from wells, and 200 μL of 4% formaldehyde was added to fix the cells for 30 minutes. Cells were washed and permeabilized with 0.1% Triton X-100 for 15 minutes. Wells were blocked with 200 μL of blocking solution (4% BSA with 0.1% Tween 20) for 1 hour. After 3 washes in PBS with 0.05% Tween 20 (PBST), wells were added to 100 μL of rabbit polyclonal anti–SARS-CoV N antibody (Rockland, 200-401-A50) at a 1:8000 dilution in dilution buffer (PBS with 0.2% Tween 20, 0.1% BSA, and 0.5% NP-40) for 1 hour. Plates were then washed 6 times in PBST and added to 100 μL of goat anti-rabbit IgG (Abcam, ab6721) at a 1:8000 dilution for 1 hour. After 6 washes in PBST, plates were developed with TMB and stopped with 0.15 M H_2_SO_4_. OD values read at 450 nm were then used to calculate percentage neutralization with the following formula: (“virus+cells” – “sample”) ÷ (“virus+cells” – “cells only”) × 100. IC_50_ values were determined using 4-parameter nonlinear regression in GraphPad Prism with curve fits constrained to have a minimum of 0% and maximum of 100% neutralization.

### Bio-layer interferometry

The affinity and kinetic constants of selected RBD variants to the ACE-2 receptor were measured by BLI performed on the Octet Red instrument (FortéBio). Assays were performed in black, 96-well plates at 25°C with agitation at 1000 rpm. Streptavidin Sensors (FortéBio) were hydrated for 20 minutes in kinetic buffer 0.01 M HEPES, 0.15 M NaCl, 3 mM EDTA, and 0.005% v/v Surfactant P20, pH 7.4 (GE Healthcare), prior to loading of 3 μg/mL of biotinylated ACE2 ligand for 180 seconds. Following loading, the baseline signal was recorded for 120 seconds in kinetic buffer. Sensors were then immersed into wells containing 2-fold serial dilutions of each recombinant SARS-CoV-2 RBD variant in kinetic buffer for 150 seconds (association phase) starting at 100 nM; this was followed by immersion in kinetic buffer for 360 seconds (dissociation phase). Curve fitting was performed using a global fit 1:1 binding model using Octet Data Analysis software v12.0.2.3 (FortéBio), and baseline drift was corrected by reference subtracting the shift of an ACE2-loaded sensor immersed in kinetic buffer only. Mean kinetic constant values from 2 independent experiments were determined, with all binding curves matching the theoretical fit with an *r*^2^ value of more than 0.99. The complex *t_1/2_* in seconds was calculated using the formula: *t_1/2_* = 1n2/k_dis_ = approximately 0.69/ k_dis_.

### Data and materials availability

All relevant data methods are available in the manuscript or in the supplemental materials.

### Statistics

All statistical and nonlinear regression analysis was performed with GraphPad Prism v9.0. The arithmetic mean of replicate neutralization measurements and the arithmetic mean of replicate measurements in other assays were used in the correlation (Spearman’s) and regression analyses for other measurements. Maximal ACE2 binding MFI was determined by the mean (quadruplicate) buffer only, i.e., ACE2 only (no inhibitor) controls. Percentage ACE2 binding inhibition was calculated as ACE2 inhibition (%) = (1 – sample ACE2 MFI/maximal ACE2 MFI) × 100. All data generated from multiplex assays represent the mean of values from experiments repeated independently twice. Throughout the manuscript, significance was defined as *P* < 0.05.

### Study approval

Study protocols were approved by the University of Melbourne Human Research Ethics Committee (2056689), Parkville, Melbourne, Australia. All associated procedures were carried out in accordance with approved guidelines, with all participants providing written informed consent in accordance with the Declaration of Helsinki.

## Author contributions

EL and AWC drafted the original manuscript. EL, ERH, KS, and AWC designed experimental protocols. EL, ERH, AA, MTO, FLM, WSL, KJS, and SKD performed experiments. AKW, PP, JAJ, SJR, NAG, SLG, LH, DFJP, DIG, and WHT contributed unique reagents. SJK and JAJ contributed unique samples. EL and AWC analyzed data. EL and ERH generated final figures. AWC conceived and supervised the study. All authors reviewed the manuscript.

## Supplementary Material

Supplemental data

## Version 1 07/12/2021

In-Press Preview

## Version 2 08/23/2021

Electronic publication
