# Supplementary material for: Simultaneous evaluation of antibodies that inhibit SARS-CoV-2 variants via multiplex assay
Source: JCI Insight. 2021 Aug 23;6(16):e150012. doi: 10.1172/jci.insight.150012 (PMC8409985; doi:10.1172/jci.insight.150012)
Supplement: Supplemental data [file jciinsight-6-150012-s063.pdf]

Supplementary Figures

Fig S1.

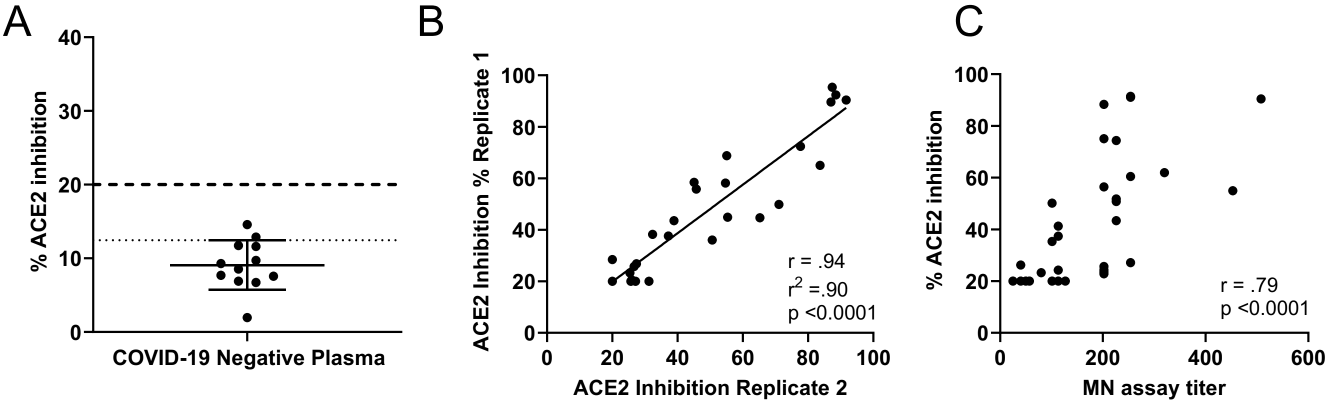

Supp Figure 1. **A**. Determination of the ACE2-RBD inhibition cut-off of 20% based of testing *n*=12 SARS-CoV-2 negative samples at a 1 in 100 plasma dilution, with the light dotted line representing 1 standard deviation from the mean, and the solid dotted line representing the assay cut-off at the Mean + 2 Standard Deviations **B.** Pearson's correlation and linear regression between the mean % inhibition obtained from testing convalescent SARS-CoV-2 samples on two independent days, by two operators **C.** Spearman's correlation of the mean % ACE2 inhibition obtained from two independent multiplex runs vs the microneutralization titer obtained for each sample

Fig S2.

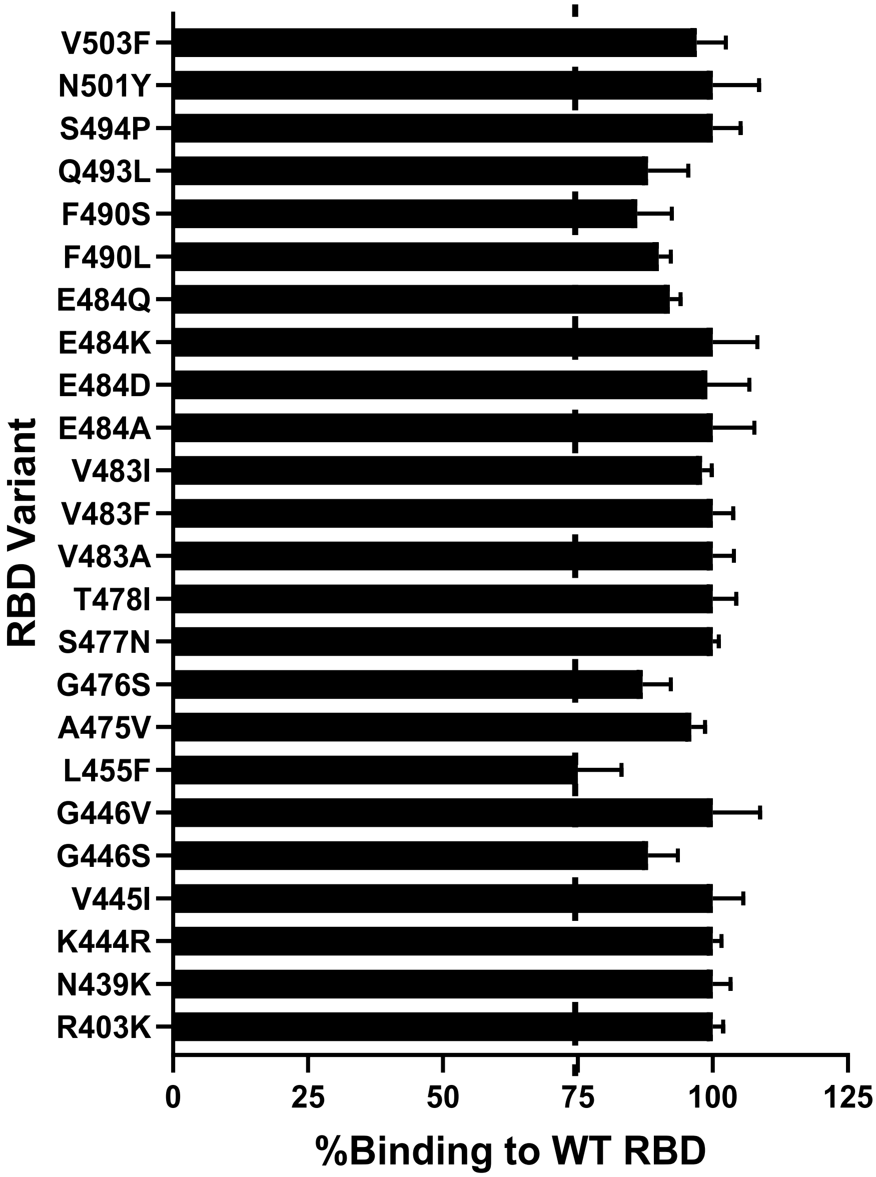

Supp Figure 2. Coupling Efficiency of RBD variants
Coupling efficiency of each recombinant RBD variant was measured on the multiplex assay with 10μg/ml of anti-His Tag antibody. Coupling of each variant was compared to the WT RBD. All included variants were coupled to at least 75% relative to WT as indicated by the dotted line. Variants with a calculated >100% coupling efficiency to WT are capped to 100%. Bars represent mean± SD, determined from two independent experiments.

Fig S3.

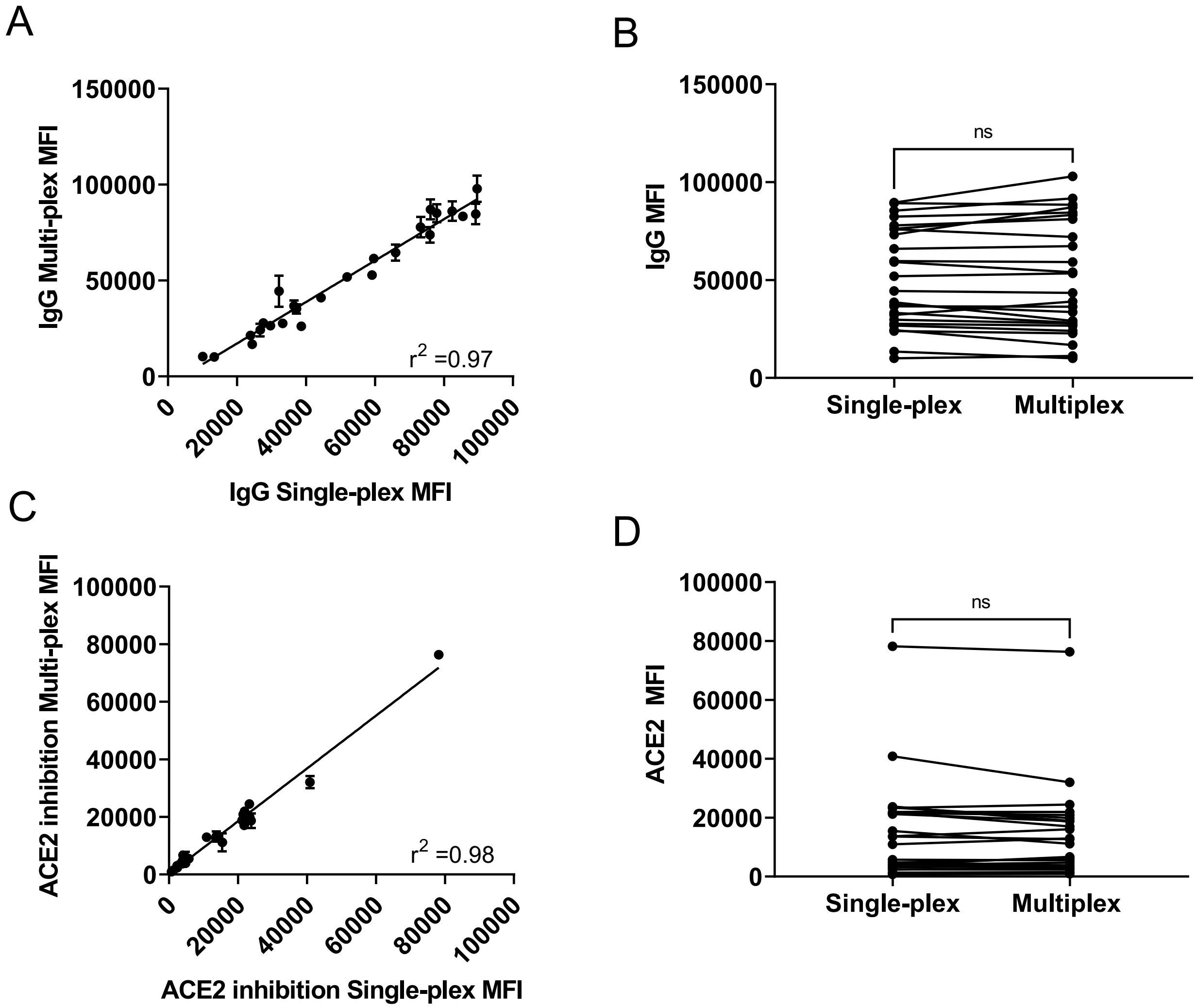

Supp Figure 3. Single-plex vs Multi-Plex Comparison

MFI values of wells containing a single bead region coupled to an RBD variant were assayed and compared to MFI values obtained for the full array of RBD variants in a single well (multi-plex). Data represent the mean of duplicates and ± SEM. **A.** Linear regression between IgG Single-plex vs Multi-plex MFI **B.** Wilcoxon paired t-test comparison between IgG Single-plex vs Multi-plex MFI**. C.** Linear regression between ACE2 inhibition Single-plex vs Multi-plex MFI. **D.** Wilcoxon paired t-test comparison between ACE2 Single-plex vs Multi-plex MFI

Fig S4.

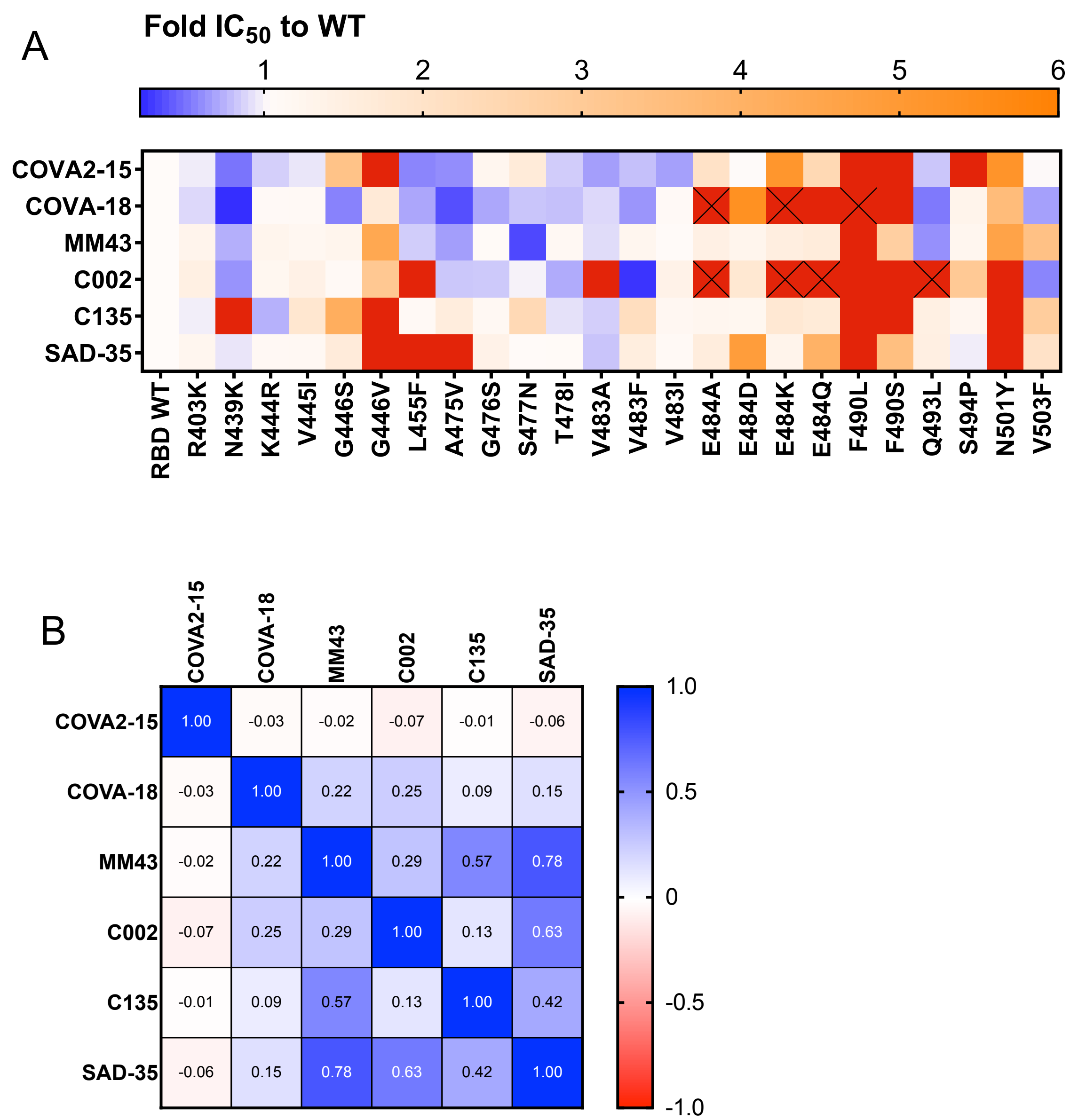

Supp Figure 4.

A. Non-Competitive assay values of RBD natural variant ACE2-RBD IC_{50} inhibition relative to RBD WT. Blue - Stronger inhibition of RBD variants relative to WT (IC_{50} < RBD WT) Orange- Weaker inhibition of RBD variants relative to WT (>1-6 fold RBD WT IC_{50}). Red >6 fold weaker RBD WT IC_{50} (Very poor/absence of inhibition)

B. Correlation matrix of RBD natural variant ACE2-RBD IC_{50} inhibition using the competitive assay format. Values reported are Pearson's r

Fig S5.

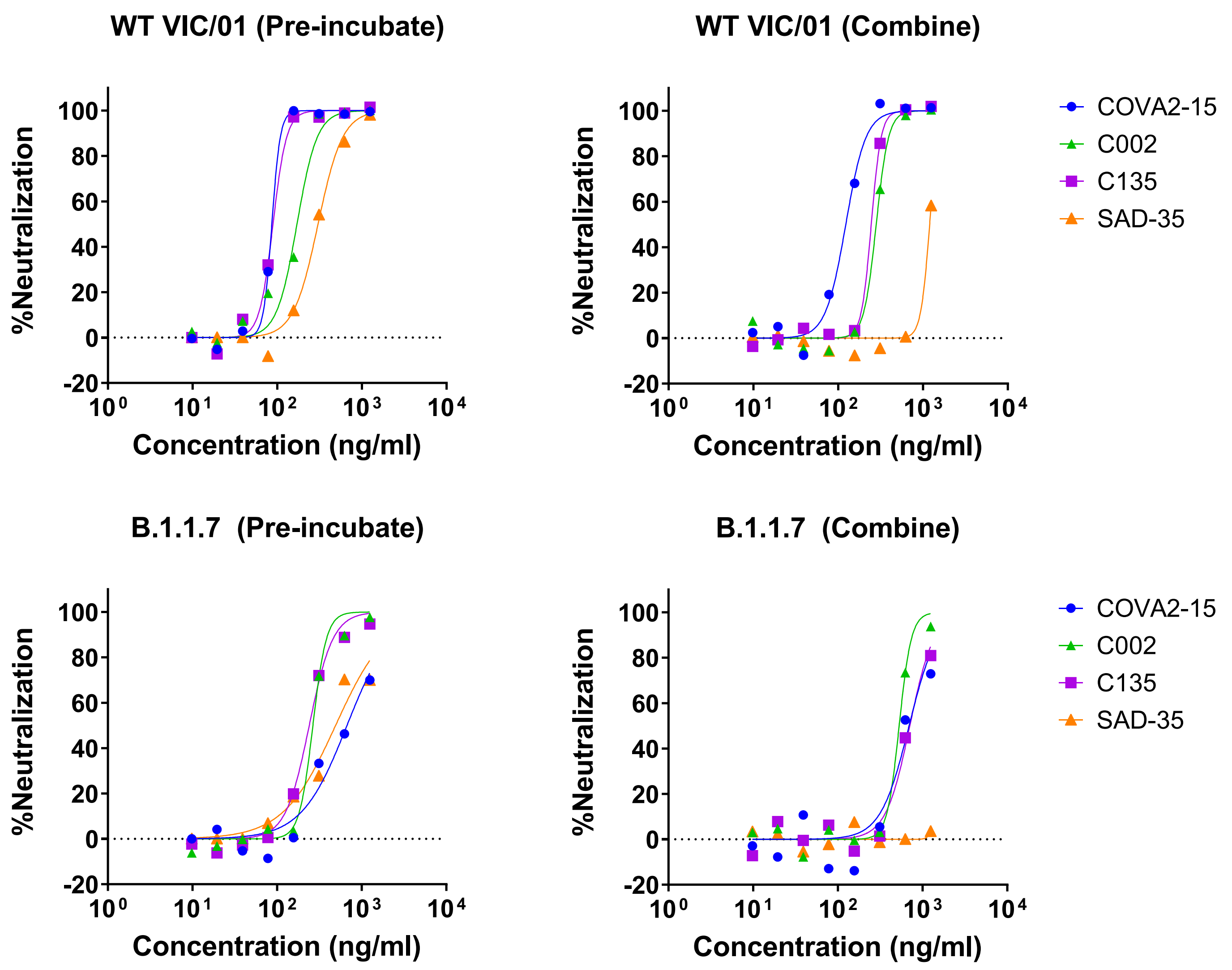

Supp Figure 5.
Graphs showing % Neutralization of WT VIC/01 and B.1.1.7 virus either pre-incubated with mAb for 1 hour vs mAb and virus added to ACE2 expressing cells together (combined)

Fig S6.

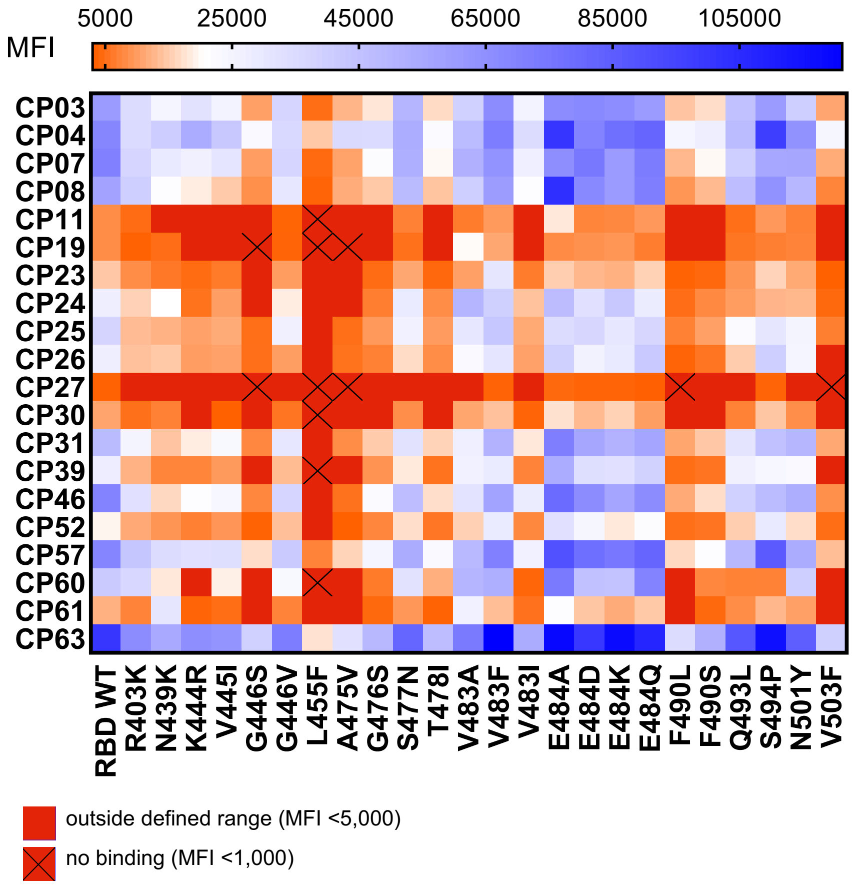

Supp Figure 6. Heat Map of Mean IgG Binding (MFI) of polyclonal convalescent plasma samples to each respective RBD variant.
Blue – High level of IgG Binding to RBD variant Orange- Weak Binding, Outside defined range = Red-Very Low Level of IgG Binding RBD variant (MFI <5,000). No binding to RBD Variant (MFI <1,000)

Table S1.

Characteristics of subjects recovered from COVID-19

Characteristic	
Age, median-years	59
Male sex-no.%	18 (85%)
Time since +nasal swab SARS-CoV2 PCR, Median*	36
Date of SARS-CoV2 PCR test	March-April 2020
Location of test	Melbourne, Victoria, Australia
Illness severity** Mild-no.(%)	8 (40%)
Moderate-no.(%)	6 (30%)
Severe-no.(%)	3 (15 %)

One subject has a false negative PCR result
**Illness severity was classified as:
Mild: prominent upper respiratory tract symptoms and not hospitalised.
Moderate: prominent lower respiratory tract symptoms and not hospitalised.
Severe: prominent lower respiratory tract symptoms and requiring hospital care.
-no data availability on illness severity of 3 subjects

Table S2.

Multiplex Assay Validation Parameters

Sensitivity	97.1% (34 of 35 MN positive)
Specificity	90.9% (10 of 11 MN Negative)
Accuracy	95.7% (44/46 MN total)
Precision	intra-% CV=1.2 *(n=6)* , inter-% CV=2.1 *(n=6)*
Robustness	r^2 0.9
Range	20-100% inhibition
Acceptance Criteria	Mean Duplicate CV <30%

Table S3

RBD Variant – ACE2 Inhibition Multiplex Assay Bead Cocktail

Bead Supplier	Catalogue #	Bead region #	Protein	Protein description	Protein supplier	Protein expression system	Protein coupled / 1x10^7 beads
Biorad	C10013-01	13	RBD-WT	SARS-CoV-2 RBD Wild-type	Genscript Plasmid	HEK293 cells	100ug
Biorad	C10072-01	72	R403K	SARS-CoV-2 RBD mutant	Genscript Plasmid	HEK293 cells	100ug
Biorad	C10043-01	43	N439K	SARS-CoV-2 RBD mutant	Genscript Plasmid	HEK293 cells	100ug
Biorad	C10047-01	47	K444R	SARS-CoV-2 RBD mutant	Genscript Plasmid	HEK293 cells	100ug
Biorad	C10054-01	54	V445I	SARS-CoV-2 RBD mutant	Genscript Plasmid	HEK293 cells	100ug
Biorad	C10063-01	63	G446S	SARS-CoV-2 RBD mutant	Genscript Plasmid	HEK293 cells	100ug
Biorad	C10073-01	73	G446V	SARS-CoV-2 RBD mutant	Genscript Plasmid	HEK293 cells	100ug
Biorad	C10014-01	14	L455F	SARS-CoV-2 RBD mutant	Genscript Plasmid	HEK293 cells	100ug
Biorad	C10020-01	20	A475V	SARS-CoV-2 RBD mutant	Genscript Plasmid	HEK293 cells	100ug
Biorad	C10026-01	26	G476S	SARS-CoV-2 RBD mutant	Genscript Plasmid	HEK293 cells	100ug
Biorad	C10046-01	46	S477N	SARS-CoV-2 RBD mutant	Genscript Plasmid	HEK293 cells	100ug
Biorad	C10044-01	44	T478I	SARS-CoV-2 RBD mutant	Genscript Plasmid	HEK293 cells	100ug
Biorad	C10066-01	66	V483A	SARS-CoV-2 RBD mutant	Genscript Plasmid	HEK293 cells	100ug
Biorad	C10051-01	51	V483F	SARS-CoV-2 RBD mutant	Genscript Plasmid	HEK293 cells	100ug
Biorad	C10056-01	56	V483I	SARS-CoV-2 RBD mutant	Genscript Plasmid	HEK293 cells	100ug
Biorad	C10067-01	67	E484A	SARS-CoV-2 RBD mutant	Genscript Plasmid	HEK293 cells	100ug
Biorad	C10019-01	19	E484D	SARS-CoV-2 RBD mutant	Genscript Plasmid	HEK293 cells	100ug
Biorad	C10022-01	22	E484K	SARS-CoV-2 RBD mutant	Genscript Plasmid	HEK293 cells	100ug
Biorad	C10015-01	15	E484Q	SARS-CoV-2 RBD mutant	Genscript Plasmid	HEK293 cells	100ug
Biorad	C10039-01	39	F490L	SARS-CoV-2 RBD mutant	Genscript Plasmid	HEK293 cells	100ug
Biorad	C10042-01	42	F490S	SARS-CoV-2 RBD mutant	Genscript Plasmid	HEK293 cells	100ug
Biorad	C10045-01	45	Q493L	SARS-CoV-2 RBD mutant	Genscript Plasmid	HEK293 cells	100ug
Biorad	C10053-01	53	S494P	SARS-CoV-2 RBD mutant	Genscript Plasmid	HEK293 cells	100ug
Biorad	C10062-01	62	N501Y	SARS-CoV-2 RBD mutant	Genscript Plasmid	HEK293 cells	100ug
Biorad	C10065-01	65	V503F	SARS-CoV-2 RBD mutant	Genscript Plasmid	HEK293 cells	100ug
Biorad	C10084-01	84	Spike-1	SARS-CoV-2 Spike-1	Sinobiological (#40591-V08H)	HEK293 cells	100ug

Table S4

Reagents used in the RBD Multiplex Bead Assay

Reagent	Multiplex Assay	Conjugate	Reagent Conc (Vol added)/well	Source	Catalogue #
RBD-Variant Multiplex Bead Cocktail	Both RBD-IgG binding & RBD-ACE2 inhibition	See Table S2	700 beads (20µl)	See Table S2	See Table S2
AviTagged Human ACE2	RBD-ACE2 inhibition	Biotin	25µg/ml (20µl)	pHLSec expression plasmid; expressed in HEK293 cells	In house- see methods
Streptavidin, R-Phycoerythrin	RBD-ACE2 inhibition	Phycoerythrin	4µg/ml (40µl)	Thermo Fisher Scientific	S866
R-Phycoerythrin, Biotin-XX Conjugate	RBD-ACE2 inhibition	Phycoerythrin	10µg/ml (10µl)	Thermo Fisher Scientific	P811
Anti-human IgG Fc	RBD-IgG binding	Phycoerythrin	1.3µg/ml (25µl)	Southern Biotech	9040-09
